# Bottom-Up and Top-Down Mechanisms of General Anesthetics Modulate Different Dimensions of Consciousness

**DOI:** 10.3389/fncir.2017.00044

**Published:** 2017-06-20

**Authors:** George A. Mashour, Anthony G. Hudetz

**Affiliations:** ^1^Department of Anesthesiology, University of MichiganAnn Arbor, MI, United States; ^2^Center of Consciousness Science, University of MichiganAnn Arbor, MI, United States; ^3^Neuroscience Graduate Program, University of MichiganAnn Arbor, MI, United States

**Keywords:** awareness, wakefulness, unconsciousness, anesthesia, sleep, anesthetic mechanisms, connectivity

## Abstract

There has been controversy regarding the precise mechanisms of anesthetic-induced unconsciousness, with two salient approaches that have emerged within systems neuroscience. One prominent approach is the “bottom up” paradigm, which argues that anesthetics suppress consciousness by modulating sleep-wake nuclei and neural circuits in the brainstem and diencephalon that have evolved to control arousal states. Another approach is the “top-down” paradigm, which argues that anesthetics suppress consciousness by modulating the cortical and thalamocortical circuits involved in the integration of neural information. In this article, we synthesize these approaches by mapping bottom-up and top-down mechanisms of general anesthetics to two distinct but inter-related dimensions of consciousness: level and content. We show how this explains certain empirical observations regarding the diversity of anesthetic drug effects. We conclude with a more nuanced discussion of how levels and contents of consciousness interact to generate subjective experience and what this implies for the mechanisms of anesthetic-induced unconsciousness.

## Introduction

Controversy persists regarding the precise mechanism of anesthetic-induced unconsciousness. A systems neuroscience approach that originated in the 1990s supports the view that anesthetics co-opt the mechanisms that have evolved to control sleep-wake cycles, suggesting a “bottom-up” cascade that results in general anesthesia (Lydic and Biebuyck, 1994; Nelson et al., 2002; Franks, 2008). More recently, network approaches to the question have suggested that disruptions of functional or effective connectivity are an agent-invariant mechanism that impairs efficient information transfer in the cortex (Casali et al., 2013; Lee et al., 2013), which—according to several theoretical frameworks—would result in unconsciousness (Hudetz and Mashour, 2016). Ketamine has played a central role in building an argument for this “top-down” approach because it activates arousal promoting centers and enhances high-frequency cortical activity but still disrupts functional connectivity in key cortical networks (Mashour, 2014).

The clear distinction or isolation of bottom-up and top-down mechanisms is almost certainly artificial, given the widespread effects of general anesthetics on both cortical and subcortical neurons as well as the dynamic signaling relationships of cortical and subcortical networks. However, there has been no theoretical framework that has effectively integrated these two perspectives of anesthetic-induced unconsciousness. Here we provide such a framework by mapping these mechanisms onto two distinct and dissociable dimensions of consciousness: *levels* and *contents* (Laureys, 2005; Overgaard and Overgaard, 2010; Bachmann, 2012; Northoff, 2013; Bachmann and Hudetz, 2014). We describe how bottom-up, subcortical mechanisms of general anesthetics depress levels of consciousness while top-down, cortical mechanisms of general anesthetics degrade the contents of consciousness. We then demonstrate how this new approach can resolve ostensibly opposing viewpoints and explain a number of phenomena that have been observed experimentally and clinically. To illustrate this, we discuss different anesthetic drugs that have more dominant effects on one or the other of these pathways. We conclude by discussing how these processes are not entirely separable but probably interact.

## Bottom-Up and Top-Down Mechanisms of General Anesthesia

It is widely accepted that the neural mechanisms controlling sleep and wakefulness are mediated by subcortical nuclei in the hypothalamus and brainstem, with the ventrolateral preoptic nucleus (VLPO) playing a key role in sleep generation. VLPO, which is active during sleep (Sherin et al., 1996), was an attractive candidate as a target for general anesthetics. Indeed, one of the first major systems neuroscience studies of anesthetic-induced unconsciousness focused on the metabolic activation of VLPO and related structures, finding neural activity patterns that were consistent with sleep (Nelson et al., 2002). Importantly, the halogenated ether isoflurane has been shown to directly activate sleep-promoting neurons within VLPO (Moore et al., 2012). The fact that a potent general anesthetic turns on a population of neurons that are active during sleep is provocative evidence of shared circuitry between physiological and pharmacological unconsciousness. In recent years, probing the shared circuits of sleep and anesthesia has been accomplished with increasingly advanced technical approaches, further supporting the hypothesis. As one example, Zhang et al. (2015) used sophisticated pharmacogenetic techniques to show that hypothalamic α2 adrenergic receptors mediate the sedative action of dexmedetomidine in a way that closely resembles recovery sleep.

The emerging scientific framework of general anesthetics modulating sleep-promoting regions is complemented by the anesthetic depression of arousal-promoting nuclei (for review (Brown et al., 2011; Leung et al., 2014)). These nuclei include the locus ceruleus (noradrenergic), pontine reticular formation (arousal-promoting GABAergic neurons), pedunculopontine and laterodorsal tegmentum (cholinergic), ventral tegmental area (dopaminergic), perifornical area (orexinergic), tuberomammillary nucleus (histaminergic) and basal forebrain (arousal-promoting cholinergic neurons). All of these regions have been demonstrated to be: (1) modulated by anesthetics in a way that would depress brain function; or (2) critical in arousal during, or emergence from, states of anesthetic-induced unconsciousness. Additionally, there is strong evidence that the thalamus plays a critical role in both sleep- and anesthetic-induced unconsciousness (Baker et al., 2014). Virtually all sedative-hypnotic drugs (with the notable exception of ketamine) metabolically depress the thalamus (Alkire and Miller, 2005), which plays multiple roles in the generation of conscious experience. The thalamus is the target for most incoming sensory information, is part of an ascending arousal system, and is also thought to coordinate cortical communication and computation (Liu et al., 2013; Mashour and Alkire, 2013). Furthermore, the rich interconnectivity of the thalamus and the cortex means that changes in thalamic activity can result in altered cortical and thalamocortical oscillations (Ching et al., 2010; Vijayan et al., 2013; Ching and Brown, 2014), with the potential for a disruption of normal information processing. Sensory-related nuclei of the thalamus may play distinct roles in modulating the level and contents of consciousness. Although the first-order sensory relays remain responsive under anesthesia, their transmission bandwidth may be reduced (Longmuir and Pashko, 1976), thus altering the contents of consciousness. Higher-order nuclei such as the pulvinar, involved in the corticocortical feedforward relay of sensory information (Sherman, 2005; Panagiotaropoulos et al., 2014; Kanai et al., 2015), may be more affected to distort conscious contents. Finally, the intralaminar nuclei play an important modulatory role in facilitating cortical arousal, information transmission and consciousness (Saalmann, 2014; Kundishora et al., 2017) and have been shown to play a key role in anesthetic sedation (Liu et al., 2013) and its reversal (Alkire et al., 2009; Baker et al., 2014). Formerly proposed to act as a consciousness switch (White and Alkire, 2003), the thalamus should be more appropriately viewed as a multidimensional controller able to modulate conscious level and content in a specific, yet interdependent manner. Collectively, there is compelling evidence that the brainstem and diencephalon support normal consciousness and that alterations in key structures within these brain regions can contribute to the generation of sleep or anesthesia.

Despite the obvious importance of bottom-up processes in supporting wakefulness, most current theories of consciousness regard corticocortical and thalamocortical networks as central to the generation of *qualia*, i.e., the subjective qualities that define experience. The primary sensory cortex is thought to be necessary but not sufficient for consciousness, higher-order association areas play important roles in top-down influences that shape perception, and the structural and functional connections between these areas are key drivers of the integration of neural information that defines the complex but seamless nature of our conscious experience (Koch et al., 2016; Tononi et al., 2016). Due to the role of cortical and thalamocortical networks in conscious experience, there has been an intense focus in the past two decades on how anesthetics modulate the cortex and an explosion of data in the past decade on how anesthetics affect connectivity and network patterns (for review see Hudetz and Mashour, 2016).

Functional magnetic resonance imaging (fMRI) and neurophysiological data acquired during consciousness, general anesthesia, sleep and vegetative states converge on a common theme: connectivity across cortical and thalamocortical networks appears important for consciousness and this connectivity is depressed or disrupted across physiological, pharmacological and pathological states of unconsciousness. The frontal-parietal network has been one area of focus, given its apparent role in connected consciousness (i.e., consciousness of the environment; lateral network) and disconnected consciousness (i.e., endogenous states of consciousness such as dreams; medial network; Demertzi et al., 2013). Although there is vigorous debate on the role of the prefrontal cortex in phenomenal consciousness vs. access consciousness (Koch et al., 2016)—i.e., pure experience vs. conscious information that can be used by other cognitive systems—a prefrontal cortex that is functionally disconnected (either from the thalamus or posterior cortex) is a remarkably consistent finding in multiple studies of anesthetic-induced unconsciousness. Indeed, both fMRI and electroencephalographic studies of propofol, sevoflurane and ketamine consistently show a functional breakdown in frontal-parietal connectivity and surrogates of frontal-parietal information transfer (Boveroux et al., 2010; Ku et al., 2011; Boly et al., 2012; Lee et al., 2013; Palanca et al., 2015; Bonhomme et al., 2016; Hudson and Pryor, 2016; Mashour, 2016; Pal et al., 2016; Ranft et al., 2016; Schroeder et al., 2016; Sleigh, 2016). The fact that ketamine conforms to this cortically-based framework of anesthetic-induced unconsciousness is remarkable, given its distinct effects on the molecular systems neuroscience and neurophysiological level compared to traditional GABAergic anesthetics. In fact, the consistent effects of ketamine and GABAergic drugs on the cortex suggest an agent-invariant feature (or related underlying mechanism) that might be a common mediator of general anesthesia. Although it would be reasonable to suggest that this frontal-parietal breakdown is merely the effect of bottom-up causes, ketamine represents a key counter-example because it suppresses (rather than activates) VLPO, activates (rather than suppresses) wake-promoting nuclei and increases (rather than depresses) thalamic metabolism (Mashour, 2014). Furthermore, cortical slice models without a thalamus demonstrate a direct effect of anesthetic suppression (by both etomidate and ketamine) on corticocortical connectivity (Voss et al., 2012). Finally, ketamine does not decrease cortical functional complexity (as measured by the perturbational complexity index) when compared to propofol or xenon (Sarasso et al., 2015).

There is thus compelling evidence for both bottom-up and top-down mechanisms of anesthetic-induced unconsciousness. These lines of investigation have been largely isolated without: (1) definitive evidence to suggest that one or the other approach is superior (although such evidence may one day be generated); or (2) a comprehensive framework that reveals the inter-relationship of the two approaches. We suggest that bottom-up and top-down actions of anesthetics modulate different dimensions of consciousness, with bottom-up processes depressing determinants of the *level of consciousness* and top-down processes degrading or disorganizing the *contents of consciousness*.

## Two Dimensions of Consciousness

Level and content are two aspects of consciousness that can be separated conceptually and empirically (Laureys, 2005). Conceptually, the level of consciousness refers to the degree to which someone is conscious, i.e., how responsive, attentive and vigilant vs. how drowsy, obtunded, or unconscious. The content of consciousness refers to what one subjectively experiences in a given moment (Dehaene and Changeux, 2011). Empirically, the level of consciousness can be assessed by the presence of eye opening, purposeful response to verbal command, reaction time, or, in some cases, a neurophysiological measure. Likewise, the content of consciousness can be empirically determined by cognitive testing, for example, by assessing the reportable awareness of sensory stimuli made perceivable or unperceivable by a suitable manipulation of stimulus properties such as contrast, duration, or masking. The level of consciousness is sometimes used synonymously with the degree of *wakefulness* or* arousal*, while the content of consciousness is used synonymously with *awareness* or *subjective experience*. Note that we cannot easily account for the possible presence of *unreportable* contents of consciousness, as the repertoire of phenomenal experiences is greater than that of reportable experiences.

There are well-known neurological conditions in which the dissociation of level and content is evident. For example, patients in a vegetative state (or, as now more properly called, unresponsive wakefulness syndrome) can display intact sleep-wake cycles with consistent eye opening and sometimes a degree of eye tracking but with no meaningful verbal expression or purposeful response. This suggests an absence of conscious content despite an apparently requisite level of wakefulness. On the other hand, normal dreaming in healthy individuals is characterized by the presence of particularly vivid mental content during periods of rapid eye movement sleep, despite strongly suppressed arousability or level of consciousness. However, as we describe in a later section, level and content cannot be completely dissociated.

## Linking Mechanisms of Anesthesia and Dimensions of Consciousness

Given the bottom-up processes that govern arousal states and the top-down processes that generate conscious content, there is no need for mutual exclusivity between the bottom-up and top-down approaches to the neural mechanisms of anesthetic-induced unconsciousness. Just as consciousness has (at least) two essential dimensions, so too can general anesthesia. The anesthetized state could be achieved by critically depressing the level of consciousness, critically degrading the contents of consciousness, or both. The general anesthetics used in routine clinical practice are likely effective because they modulate both levels and contents of consciousness through, respectively, bottom-up and top-down mechanistic pathways.

There are several advantages to this theoretical framework for the mechanism of anesthetic-induced unconsciousness. First, it helps integrate two lines of investigation that have been largely disconnected. Although it has likely been implicitly recognized that there is an artificial dichotomy between the bottom-up and top-down approaches to anesthetic mechanism, there has been no explicit synthesis of these lines of investigation and the associated neurobiology. Resolving the controversy through a broader and well-established paradigm for thinking about consciousness creates the potential for new synergies and more meaningful synthesis of data on anesthetic-induced unconsciousness.

A multidimensional framework for the mechanism of anesthetic-induced unconsciousness also explains several observations. For example, it has been consistently demonstrated that there is a widespread disruption of corticocortical network connectivity upon induction of anesthesia, but there is limited return of corticocortical connectivity upon initial recovery from anesthesia, with subcortical regions showing dominant activity (Långsjö et al., 2012). The asymmetry of cortical connectivity patterns pre- vs. post-anesthesia can be seen to correlate with the asymmetrical *contents* of consciousness, which are rich just prior to the onset of anesthesia but likely impoverished upon initial recovery. Moreover, certain cognitive functional networks are more dominant during or after emergence than in preanesthetic baseline (Liu et al., 2013); emergence may depend on the brain traversing an orderly sequence of metastable activity states (Hudson et al., 2014). The considerable variability in the electroencephalographic signature of surgical patients regaining conscious awareness suggests the existence of qualitatively different emergence trajectories (Chander et al., 2014).

Furthermore, we can better appreciate the differences of sedative-hypnotic agents based on their activity profile along the axes of levels and contents of consciousness. As one example, dexmedetomidine is thought to induce a sleep-like state with rapid reversal of consciousness in response to stimuli. Of interest, dexmedetomidine appears to act primarily through bottom-up mechanisms (Akeju et al., 2014), with relative sparing of frontal-parietal network connectivity (although these regions are metabolically depressed) in contrast to studies of propofol, sevoflurane and ketamine. The preserved machinery for sustaining conscious content might allow for this reversibility, with levels being the primary functional substrate. Conversely, ketamine activates arousal-promoting nuclei, depends (in part) on wake-promoting neurotransmitters such as norepinephrine for its hypnotic action, increases cortical acetylcholine levels, and preserves signs of wakefulness such as eye opening, movement, and higher-frequency electroencephalographic activity (Lu et al., 2008; Kushikata et al., 2011; Pal et al., 2015; Li and Vlisides, 2016). As such, the level of consciousness is maintained in several respects, but the contents of consciousness become disorganized in a way that precludes processing of the environment. Loss of connected consciousness occurs despite evidence for intact representation of environmental events in somatosensory cortex or even disconnected conscious events such as hallucination. It could be argued that the relatively “one-dimensional” actions of dexmedetomidine (primarily modulating levels of consciousness) and ketamine (primarily modulating contents of consciousness) are what limit their use as sole agents for maintenance of surgical anesthesia. By contrast, the more profound actions of propofol and halogenated ethers could be defined by their dual effect on both levels of consciousness (mediated through subcortical sleep-wake networks) and contents of consciousness (mediated through thalamocortical and corticocortical networks).

## Discussion

We have argued that the level and contents of consciousness can be distinguished and they map on to, respectively, bottom-up and top-down information processing. In fact, most investigations to-date focused on studying either the content or level of consciousness applying a form of *contrastive analysis* (Sandberg et al., 2014) comparing, for example, the neural signatures of perceived vs. non-perceived stimuli or of responding vs. non-responding subjects.

We must acknowledge, however, that level and content may be more intertwined than generally appreciated. For example, the content of conscious experience may be different when studied at different levels of wakefulness. Moreover, as was recently argued (Bachmann and Hudetz, 2014), a certain level of consciousness is probably necessary for having any phenomenal (i.e., mental) content. In other words, one cannot be aware of anything if the level of consciousness is zero. Likewise, we could not speak of a level of consciousness in the complete absence of content. Our conceptual model leads to the prediction that the neuronal pathways and neurophysiological mechanisms that account for and modulate the level and content of consciousness are also interrelated. One could imagine that “bottom-up” and “top-down” processes interface at several, hierarchically positioned interfaces.

It is also possible that, for the purpose of objective study and clinical assessment, even the bivariate representation of consciousness, as defined in terms of level and content, will be insufficient (Northoff, 2013). Consciousness may, eventually, have to be defined in a multidimensional framework that could even include behavioral data. For example, Monti et al characterized the state of consciousness of neurological patients and healthy subjects in a three-dimensional continuum using awareness, wakefulness and mobility as the principal axes (Monti et al., 2009). This scheme allows one to distinguish the case of disconnected consciousness (Sanders et al., 2012), in which subjects are nonresponsive but mentally active (aware), from those who are fully unconscious (unaware) as well as various transitional conditions along a spectrum.

Although there are undoubtedly complexities when considering dimensions of consciousness as substrates for the mechanisms of general anesthesia, the bivariate approach that is proposed here is an important first step for integrating two lines of investigation into anesthetic mechanisms in a neurobiologically meaningful way. Mapping bottom-up anesthetic mechanisms to levels of consciousness and top-down anesthetic mechanisms to contents of consciousness enables a new approach to classifying general anesthetics (as was shown in the examples of dexmedetomidine and ketamine), can help explain clinical phenomena, and points the way to a more comprehensive systems-neuroscience approach that might shed light on the dimensionality of consciousness itself.

## Author Contributions

GAM conceived of the idea; GAM and AGH developed the ideas and wrote the manuscript.

## Conflict of Interest Statement

The authors declare that the research was conducted in the absence of any commercial or financial relationships that could be construed as a potential conflict of interest.
